# Supplementary material for: HIVprotI: an integrated web based platform for prediction and design of HIV proteins inhibitors
Source: J Cheminform. 2018 Mar 9;10:12. doi: 10.1186/s13321-018-0266-y (PMC5845081; doi:10.1186/s13321-018-0266-y)
Supplement: Supplementary file 2 — Additional file 2. Source code of HIVProtI web server. [file 13321_2018_266_MOESM2_ESM.zip › HIVprotI_Source-code/hivprotI/ic50_predict.php]

HIVprotI Pred


include("head.php");
?


#### IC_50_ Prediction

Following is the  JSME editor , through which user can draw input of its desired chemical compound, or paste input in the form of SMILES, MOL and SDF format and subsequently predict its IC_50_ on selected HIV proteins.

  

  
  


**Draw your molecule:**

Your web browser must have JavaScript enabled
in order for this application to display correctly.

  
  

| Reverse transcriptase |  | Protease |  | Integrase |  | CHECK ALL |

  

---


***Note***: The prediction will take 2-5 minutes........

include("foot.php");
?
